# Central nervous system involvement in pediatric hemophagocytic lymphohistiocytosis: A single-center descriptive study of clinical features, neurodiagnostic findings, and outcomes

**DOI:** 10.46989/001c.155719

**Published:** 2026-02-19

**Authors:** Manan Nath, Anshul Vagrecha, Annie H. Roliz, Yash D. Shah, Robin Varughese, Ramya Trietel, Alan Johnson, Carolyn Fein-Levy, Sanjeev V. Kothare

**Affiliations:** 1 Division of Pediatric Neurology, Arkansas Children’s Hospital, Little Rock, AR, USA; 2 Division of Hematology-Oncology, Cohen Children’s Medical Center, New Hyde Park, NY, USA; 3 Division of Pediatric Neurology, Cohen Children’s Medical Center, New Hyde Park, NY, USA; 4 Division of Pediatric Neurology, Our Lady of the Lake Children’s Hospital, Baton Rouge, LA, USA; 5 Division of Neuroradiology, Northwell Health, New Hyde Park, NY, USA

**Keywords:** pediatric, HLH, CNS

## Abstract

Hemophagocytic lymphohistiocytosis (HLH) is a life-threatening hyperimmune condition triggered by the pathological activation of T-cells, natural killer cells, and macrophages leading to a cytokine storm with widespread hyperinflammation affecting multiple organ systems, including the brain. This retrospective chart review characterizes the central nervous system (CNS) features in 22 pediatric HLH patients at a single center. CNS involvement was determined based on symptoms, exam, abnormal cerebrospinal fluid (CSF) studies, neuroimaging, and electroencephalogram (EEG) findings. Twenty-two children with HLH were analyzed, classifying them into primary HLH (pHLH) and secondary HLH (sHLH) based on genetic testing. Of 20 patients who underwent genetic testing, 6 (30%) had pathogenic pHLH mutations, while 14 were sHLH (8 with variants of unknown significance, 6 with no variants). CNS involvement was noted in 17 (77.2%) patients. Symptoms included generalized weakness, altered sensorium, seizures, and headaches. MRI abnormalities included patchy T2 FLAIR prolongation (85%) with and without contrast enhancement and subdural collection, diffuse brain atrophy (40%), microhemorrhages (15%) and diffusion restriction (15%) patients. CSF studies showed higher WBC counts in pHLH . Therapies amongst this cohort varied, with 47% receiving dexamethasone, etoposide and cyclosporine, and 29% undergoing HSCT. Mortality in the first year was 18%, with 75% of deaths involving patients with CNS involvement of HLH. Mean survival at six months was 167 days, with no further deaths in the 12-year follow-up. We conclude that frequent neurological abnormalities are noted in children with HLH highlighting the role for active surveillance of CNS involvement in this group of patients.

## Key Message

- This retrospective study reviewed 22 children diagnosed with hemophagocytic lymphohistiocytosis (HLH).
- It characterizes the clinical features, genetic profiles, laboratory findings, and neurodiagnostic studies in HLH patients with neurological involvement.
- Central nervous system (CNS) involvement in pediatric HLH is associated with significant morbidity and mortality.
- Implementing institutional protocols to screen children with HLH for CNS involvement may improve survival outcomes.

## Introduction

Hemophagocytic lymphohistiocytosis (HLH) is a life-threatening hyperimmune condition triggered by the pathological activation of T cells, natural killer cells, and macrophages, leading to a cytokine storm and widespread hyperinflammation. This condition often affects multiple organs, including the liver, lymph nodes, spleen, and brain.^1^

Historically considered rare, HLH has an estimated incidence of 1.2 cases per million children, with 40% of cases occurring in adults.^2–4^ Approximately 1 in 3000 patients admitted to pediatric tertiary centers may have HLH.^5^ Current diagnostic and management guidelines are based on the HLH 2004 study conducted by the Histiocyte Society.^6^ Patients must meet five out of eight diagnostic criteria established by the HLH-2004 group (Table 1).^7^ While not part of the formal diagnostic criteria, many HLH patients exhibit central nervous system (CNS) signs and symptoms.

**Table 1.** HLH Diagnostic Criteria.^6^

Homozygosity or compound heterozygosity for pHLH mutations in children
**Or**
Fever > 38.5 °C
Splenomegaly
Cytopenia > 2 cell lines: Hb <9g/dL; <10g/dL (infants) Platelets <100 x 10^9^/L Absolute neutrophil count <1.0 x 10^9^/L
Hypertriglyceridemia (Fasting triglycerides > 265 mg/dL) AND/OR hypofibrinogenemia (Fibrinogen < 150 mg/dL)
Hemophagocytosis in bone marrow, liver, spleen or lymph nodes
Low or absent NK cell activity
Ferritin > 500 ng/mL
Elevated soluble CD25 (soluble IL-2 receptor alpha [sIL-2R]) two standard deviations above age adjusted lab specific reference range

pHLH= primary hemophagocytic lymphohistiocytosis (pHLH), hemoglobin(Hb),Natural Killer(NK) ,Cluster of Differentiation 25(CD25),Interleukin 2 (IL2)

Familial HLH (fHLH), also known as primary HLH (pHLH), is caused by homozygous or compound heterozygous mutations in genes involved in the cytotoxic function of T cells.^1,8^ Mutations in PRF1, UNC13D, STX11, STXBP2, Rab27, SH2D1A, BIRC4, LYST, ITK, SLCA7, XMEN, and HPS are common causes of pHLH, with inheritance patterns being autosomal recessive or X-linked.^1,9,10^ Secondary HLH (sHLH) can be triggered by infections, malignancies, or underlying rheumatologic conditions, with Epstein-Barr virus (EBV) infection often associated with severe cases.^11–14^

CNS involvement in HLH is characterized by the aberrant activation and infiltration of inflammatory cells into the leptomeninges, brain parenchyma, and perivascular spaces, leading to a cytokine storm that can cause demyelination, parenchymal necrosis, and calcifications.^8,14–20^ Neurologic manifestations vary and may include generalized weakness, altered sensorium, headaches, seizures, cranial nerve palsies, ataxia, irritability, altered consciousness, and hypotonia.^21–25^ Neuroimaging changes may precede neurologic symptoms, though the timeline of CNS involvement remains unclear.^26^ CNS involvement occurs in 30-70% of HLH cases and can be the sole clinical presentation.^21–25^ Cerebrospinal fluid (CSF) pleocytosis and modest protein elevation are observed in 10-47% of patients.^27–30^ Neuroimaging findings include nonspecific brain atrophy, multifocal symmetric periventricular white matter abnormalities, calcifications, focal cortical/subcortical lesions, and hemorrhage.^14,18,19^

HLH can be fatal without treatment. Current therapy for pHLH includes etoposide and dexamethasone, with intrathecal methotrexate added for CNS involvement.^5,31^ Patients with identified pHLH mutations require hematopoietic stem cell transplantation (HSCT), which, if performed early, may halt CNS disease progression and prevent relapses.^5,32^ Many pediatric and adult patients with sHLH lack identifiable genetic mutations and are not candidates for HSCT.

Despite established diagnostic criteria, diagnosing HLH with CNS manifestations (CNS-HLH) remains challenging, as it can mimic conditions like acute disseminated encephalomyelitis, septic shock, malignancy, and hyperinflammatory syndromes such as multisystem inflammatory syndrome in children (MIS-C).^33–35^ Although outcomes have improved with allogeneic bone marrow transplantation,^36^ CNS-HLH continues to carry a high burden of morbidity and mortality, with motor and cognitive deficits and a 5-year survival rate of 40-67%.^16,21^ The true burden of CNS disease in sHLH and its optimal management remain unclear.

## Primary aim

This study aimed to characterize CNS features in pediatric HLH patients.

## Methods

This retrospective chart review included pediatric patients hospitalized with HLH from August 26, 2013, to September 5, 2020, at a single medical center. Patients were followed for up to 12 years. Data were collected from electronic medical records after Institutional Review Board approval.

Patients were identified using the ICD-10 code for HLH. Of 48 children, 26 were excluded for not meeting HLH-2004 diagnostic criteria or lacking a molecular diagnosis. Patients with prior HLH diagnoses not primarily hospitalized at the institution were also excluded. Inclusion criteria were pediatric patients meeting HLH-2004 diagnostic criteria, including both pHLH and sHLH cases.

Data collected included demographics, genetic test results, physician reported history of symptoms and clinical signs, CSF and blood viral panels and bacterial cultures.

Laboratory results included initial, maximum, and minimum values of hemoglobin, platelets, absolute neutrophil count, ferritin, C-reactive protein, erythrocyte sedimentation rate, aspartate transaminase, alanine transaminase, gamma-glutamyl transpeptidase, triglycerides, fibrinogen, lactate dehydrogenase, direct and indirect bilirubin, and interleukin-2 receptor antibody levels.

CNS involvement was defined by neurologic symptoms and signs, abnormal CSF studies, neuroimaging, or EEG. CSF abnormalities included leukocyte counts >5 cells/mL or protein >50 mg/dL. Brain MRI findings, reviewed by a neuroradiologist, included diffuse atrophy, T1/T2 abnormalities, diffusion restriction, micro-/large hemorrhages, or calcifications. EEG reports were reviewed for focal or generalized slowing and epileptiform abnormalities.

## Statistical analysis

Data were entered into REDCap and analyzed using IBM SPSS Statistics for MacBook (version 28.0.1.0). Categorical variables were compared using Chi-square or Fisher’s exact tests, and non-parametric continuous variables were analyzed using the Mann-Whitney U test, reported as medians with interquartile ranges. A p-value <0.05 was considered significant.

## Results

Twenty-two children were included. Please refer to **Table 2** for detailed demographic information. Genetic testing was performed in 20 patients, with 6 (30%) having pathogenic mutations in pHLH genes, classifying them as pHLH. The remaining 14 were classified as sHLH, with 8 having variants of unknown significance and 6 having no variants. The clinical characteristics and CNS findings of these pHLH patients are summarized in **Table 3**.

**Table 2.** Demographic information.

		**Pediatric patients N = 22**
Female : Male		14 : 8
Age <12 months		6/22 (27.3%)
Age 12 months to 18 years		16/22 (72.7%)
Race	Caucasian	8 (36.4%)
	African American	4 (18.2%)
	Hispanic	3 (13.6%)
	South Asian	4 (18.2%)
	Other*	3 (13.6%)

*Other ethnicities comprising Jewish, Arab, African, Eastern European and Russian

**Table 3.** Summary of children with primary HLH with pathogenic genetic mutations.

	**Gene mutation**	**Age**	**Gender**	**Ethnicity**	**CNS symptoms**	**MRI abnormality**	**CSF Labs**	**EEG**	**Patient outcome**
Patient 1	Compound heterozygous frameshift PRF1 mutation in c.50del(p.Leu17fs) and c.985dup(p.Val329fs)	23 days	Male	African American	Generalized weakness, altered sensorium, irritability	T1T2 gray matter changes; left perirolandic stroke; microbleeds	CSF WBC 8, CSF Protein 67	Generalized slowing	Deceased
Patient 2	Homozygous pathogenic STXBP2 partial deletion (19p13.2(7709251-7713461)	1 month	Male	African American	Generalized weakness, altered sensorium, seizure-like activity	T1T2 white matter contrast enhanced; cerebral atrophy; left subdural collection; punctate microhemorrhages	CSF WBC 1, CSF Protein 20.4		
Patient 3	Pathogenic UNC13D inversion	3 months	Female	Hispanic	Generalized weakness	Cerebral atrophy	CSF WBC 9, CSF Protein -		
Patient 4	Heterozygous missense pathogenic UNC13Dc.904C>T,(p.Leu302Phe), MEFV c.586G>T,(p.Gly196Trp), SH2D1AchrX:123480246(c-247G>A)	9 months	Male	African American	Seizure-like activity	T1T2 gray matter changes, Acute infarcts at 3months age that resolved subsequently	CSF WBC 22, CSF Protein 33.2		
Patient 5	Heterozygous pathogenic STX11(c.173T>C,p.Leu58Pro)	23 months	Male	South Asian	Focal weakness, vision abnormality	T1T2 white matter contrast enhanced. Optic nerve sheath enhancement. Diffusion restriction. Subdural collection	CSF WBC 43, CSF Protein 61.1	Generalized slowing	Deceased
Patient 6	Homozygous pathogenic STX11 p.Gln230Alafs*125	29 months	Male	South Asian	Altered sensorium	T1T2 gray matter changes	CSF WBC 744, CSF Protein 75.8		

Cerebrospinal fluid (CSF), White blood cell (WBC), WBC as cells/µL, Protein as mg/dL

Four (18.2%) children had coexisting malignancies. No significant differences were observed in infection, sepsis, meningitis, multi-organ dysfunction, stroke, or hematologic/rheumatologic abnormalities between survivors and deceased patients. Details regarding coexisting conditions can be seen in **Table 5.**

**Table 4.** Summary of deceased (n=3) HLH patients with Central Nervous System involvement

**Gene mutation**	**Demographic information**	**CNS symptoms**	**Systemic labs**	**CSF Labs**	**MRI**	**EEG**	**Therapy**	**Outcome**
Compound heterozygous frameshift PRF1 mutation in c.50del(p.Leu17fs) and c.985dup(p.Val329fs)	23 days old African American male	Weakness Altered sensorium, irritability	Hb 9.7, Pl 76, Ferritin 30000, CRP 37, LDH 1232, IL2r 25000	CSF WBC 8, CSF Protein 67	T1T2 gray matter changes, microbleeds	Generalized background slowing	Anakinra, dexamethasone, etoposide, IVIG, HSCT	Deceased within 4 months from presentation
Heterozygous pathogenic STX11(c.173T>C,p.Leu58Pro)	23 months South Asian male	Focal weakness vision abnormality	Hb 7.5, Pl 21, Ferritin 15500, CRP 37, LDH 849, IL2r 7088	CSF WBC 43, CSF Protein 61.1	T1T2 white matter contrast enhanced Optic nerve sheath enhancement Diffusion restriction Subdural collection	Generalized background slowing	Anakinra, dexamethasone, etoposide, methotrexate, IVIG, HSCT	Deceased within 7 months from presentation
LYST variation of uncertain significance	6.5 year old African American female	Generalized weakness	Hb 4, Pl 22, Ferritin 5100, CRP 189, LDH 1800, IL2r 13429	CSF WBC 1, CSF protein 62	No MRI changes	Generalized background slowing	Anakinra, dexamethasone	Deceased within 1 month from presentation

(Hb) Hemoglobin, (CSF) cerebrospinal fluid, (WBC) White blood cell, (CRP) C-reactive protein, (LDH) Lactate dehydrogenase, (IVIG) Intravenous immunoglobulin, (HSCT) Hematopoietic stem cell transplant, Hb as g/dL, Pl as ×10^3/µL, Ferritin as ng/mL, CRP as mg/L, LDH as U/L, sIL-2R as pg/mL, WBC as cells/µL, Protein as mg/dL

**Table 5.** Coexisting conditions in patients with HLH (N = 22)

Non-specific infection	12 (54.5%)
EBV	6 (27.2%)
CMV	2 (9%)
Malignancy	4 (18.2%)
Sepsis	9 (40.9%)
Meningitis	1 (4.5%)
ADEM	1 (4.5%)
Multi-organ dysfunction	2 (9%)
Stroke	0
DIC	2 (9%)
MAS	1 (4.5%)

ADEM: acute disseminated encephalomyelitis; CMV: cytomegalovirus; DIC: disseminated intravascular coagulation; EBV: Epstein-Barr virus; MAS: macrophage activation syndrome

17 (77.2%) were noted to have CNS involvement. Neurology consultations were obtained for 8 (36.3%) patients at a median of 26 days from symptom onset.

Seventeen (77.2%) patients developed CNS symptoms during hospitalization. Symptoms were overlapping and included generalized weakness 11 (30.3%), altered sensorium 8 (47%), seizure/seizure-like activity 4 (23.5%), headaches 3 (17.6%) and focal weakness with vision abnormalities 1 (5.8%).

**Figure 1.**
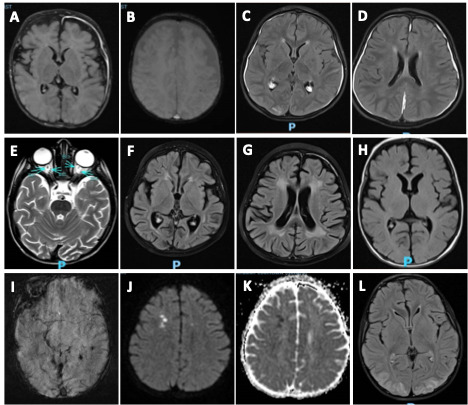
MRI abnormalities in pediatric patients with primary HLH. **A,B)** 1-month old boy with heterozygous STXBP2 mutation with initial normal MRI and subsequent post contrast axial T2 FLAIR imaging showing mild cerebral atrophy with left cerebral subdural effusion. Susceptibility weighted image shows punctate petechial hemorrhage. **C-E)** 23-month-old boy with homozygous pathogenic STX11 mutation with initial post-contrast axial T2 FLAIR images showing subdural fluid collection, right occipital dural enhancement and optic nerve sheath enhancement. **F, G)** 2 months later, post contrast axial T2 FLAIR images show cerebral volume loss and white matter T2 prolongation. **H, I)** 23-day old baby boy with compound heterozygous frameshift PRF1 mutation with axial T2 and SWI images showing left peri-rolandic gray matter involvement with focus of left temporal punctate hemorrhage. **J-L)** 3-month-old baby girl with pathogenic UNC13D mutation with DWI and ADC images showing diffusion restriction which

MRI scans were obtained in 20 of 22 (90.9%) patients at a median of 17 days from symptom onset with overlapping findings. Patchy T2 FLAIR prolongation was noted in 17 (85%) patients with 6 (35.2%) patients having T2 FLAIR prolongation in gray matter. Other MRI abnormalities included diffuse brain atrophy 8 (40%), microhemorrhage 3 (15%) and diffusion restriction 3 (15%). MRI abnormalities in pediatric pHLH patients are shown in **Figure 1**.

6 (27.2%) patients received an EEG with 5 (83.3%) patients showing mild/moderate generalized polymorphic slowing suggesting etiologically non-specific diffuse cortical dysfunction, 1 patient exhibiting interictal epileptiform discharges and 1 patient with a recorded electrographic seizure.

No significant differences were observed in laboratory values between patients with and without CNS involvement. Please refer to supplemental **Table 6** for details. CSF studies in 17 patients revealed a median WBC count of 15.5 cells/mL in pHLH compared to 1 cell/mL in sHLH (p=0.005). Median CSF values with interquartile ranges are detailed in **Table 7.**

**Table 6.** Serum labs in HLH patients (median values with interquartile range)

	**No CNS involvement**	**CNS involvement**	**p-⁠value**
Initial Hb (g/dL)	10.1 (1.5)	9.8 (2.7)	0.703
Initial Platelets ×10^3/µL	128.5 (132)	61 (148)	0.521
Initial ANC cells/µL	2.4 (4.13)	1.4 (8.0)	0.810
Maximum Ferritin (ng/ml)	14465 (19960)	6120 (22858)	0.897
Maximum CRP (mg/dL)	8.4 (47.8)	37.7 (145.9)	0.172
Maximum ESR	34.5 (39)	38.5 (67)	0.554
Maximum AST (U/L)	1121 (1519)	374 (794)	0.144
Maximum ALT (U/L)	686 (432)	338 (754)	0.172
Maximum GGT	185 (-)	137 (296)	0.641
Maximum Triglycerides (mg/dL)	561 (588)	586 (574)	0.829
Minimum Fibrinogen	131.5 (320.5)	179 (641)	0.763
Maximum LDH	2357 (1244)	1323 (981)	0.237
Maximum Direct Bilirubin (mg/dL)	0.45 (1.1)	1.54 (4.3)	0.290
IL2-receptor antibody	7682 (9228)	8916 (17171)	1.000

C-reactive protein (CRP), erythrocyte sedimentation rate (ESR), aspartate transaminase (AST), alanine transaminase (ALT), gamma glutamyl transpeptidase (GGT),

**Table 7.** CSF labs in HLH patients (median values with interquartile range)

	**No CNS symptoms**	**CNS symptoms**	**p-value**
CSF WBC (cells/μL)	1 (-)	1.5 (11)	0.900
CSF RBC (cells/μL)	289 (-)	3 (119)	1.000
CSF protein (mg/dL)	-	40.4 (39.8)	0.923
CSF glucose (mg/dL)	-	59.5 (17)	0.154

Cerebrospinal fluid (CSF), White blood cell (WBC), Red blood cell (RBC)

Of 17 patients with CNS involvement, 8 (47.05%) received dexamethasone, etoposide, and cyclosporine; 6 (35.2%) received dexamethasone alone; 2 (11.7%) received intrathecal methotrexate; and 5 (29.4%) underwent HSCT. Other treatments included IV immunoglobulins, anakinra, anti-thymocyte globulin, and rituximab.

In the initial one year, 4 (18%) children died, with 3 (75%) having CNS involvement. Mean survival duration at six months was 167 days. No further deaths occurred during the 12-year follow-up. See **Table 4** for details regarding deceased HLH patients with CNS involvement).

## Discussion

This study describes CNS features in 22 pediatric HLH patients over seven years. CNS involvement was determined by neurologic symptoms and abnormal neurodiagnostic studies, with 77% of patients demonstrating CNS involvement. Generalized weakness and altered sensorium were the most common neurologic symptoms with 23% patients noted to have seizures. This is consistent with previous studies.^21–25,30,37^

CNS symptoms likely result from meningeal and parenchymal inflammation due to lymphocytic and macrophage infiltration.^38^ One patient with pHLH exhibited an acute stroke that resolved but later developed posterior reversible encephalopathy syndrome (PRES) due to HLH reactivation.

CSF findings of increased WBC counts are consistent with findings of Shyu et al.^39^ The basis of CSF pleocytosis is likely related to the aberrant neuroinflammatory response involving a dysregulated cytokine and interleukin cascade.

T2 FLAIR prolongation in gray matter was frequently observed, possibly due to impaired mitochondrial oxidative phosphorylation or global hypoperfusion.^40^ Brain atrophy, microhemorrhages, white matter abnormalities, and leptomeningeal enhancement were also noted, consistent with prior findings.^14,41^ Higher CSF WBC count in patients with pHLH compared to sHLH may be due to difference in pathophysiology. The underlying genetic mutation in pHLH can produce a more pronounced aberrant activation and infiltration of macrophages and microglia in the CNS, resulting in higher degree of CSF pleocytosis.

Treatment followed HLH 2004 protocols, with dexamethasone, etoposide, cyclosporine, and intrathecal methotrexate used for CNS involvement. HSCT was pursued early. Variability in management reflects the study’s retrospective nature and evolving practices over time.

Mortality was 18%, primarily occurring within the first nine months. Of the two patients who received HSCT and then died, one patient developed pneumonia (post HSCT) and subsequent respiratory failure leading to demise, and the other developed CMV viremia and ARDS leading to demise.

## Limitations

The retrospective design and management variability over seven years may have influenced results. Defining CNS involvement based on symptoms and neurodiagnostic studies may not capture all cases. The rarity of HLH limits generalizability, and larger studies are needed to differentiate pHLH and sHLH.

## Conclusion

This study highlights the significant CNS involvement (77%) in pediatric HLH patients. Early and aggressive management may alter the disease course. Newer therapies such as ruxolitinib may yet also alter the natural history of this disease. Treating clinicians should remain vigilant in surveilling for, recognizing and treating CNS involvement in HLH patients.

### Author Contributions

Conceptualization: Manan Nath (Equal), Anshul Vagrecha (Equal), Sanjeev V. Kothare (Equal). Methodology: Manan Nath (Equal), Anshul Vagrecha (Equal), Sanjeev V. Kothare (Equal). Formal Analysis: Manan Nath (Equal), Anshul Vagrecha (Equal), Sanjeev V. Kothare (Equal). Investigation: Manan Nath (Equal), Anshul Vagrecha (Equal), Yash D. Shah (Equal), Robin Varughese (Equal), Ramya Trietel (Equal), Alan Johnson (Equal), Carolyn Fein-Levy (Equal), Sanjeev V. Kothare (Equal). Writing – original draft: Manan Nath (Equal), Anshul Vagrecha (Equal), Carolyn Fein-Levy (Equal), Sanjeev V. Kothare (Equal). Writing – review & editing: Manan Nath (Equal), Annie H. Roliz (Equal), Carolyn Fein-Levy (Equal), Sanjeev V. Kothare (Equal). Resources: Manan Nath (Equal), Anshul Vagrecha (Equal), Carolyn Fein-Levy (Equal), Sanjeev V. Kothare (Equal). Supervision: Sanjeev V. Kothare (Lead).

### Conflict of Interest Statement

The author(s) declare(s) that there is no conflict of interest.

### Financial Disclosures

Dr. Nath, Dr. Roliz, Dr. Shah, Dr. Treitel, Dr. Varughese, Dr. Johnson, Dr. Fein-Levy and Dr. Kothare report no disclosures
